# Real-World Data of Light Chain (AL) Amyloidosis: Prognostic Indices and Treatment Patterns

**DOI:** 10.3390/biomedicines13112734

**Published:** 2025-11-08

**Authors:** Marko Mitrovic, Aleksandra Sretenovic, Natalija Kecman, Nikola Vukosavljevic, Maja Perunicic Jovanovic, Dragana Sobic Saranovic, Ruzica Maksimovic, Zoran Bukumiric, Danijela Lekovic, Arsen Ristic, Milena Todorovic Balint, Jelena Bila

**Affiliations:** 1 Clinic of Hematology, Department for Multiple Myeloma, University Clinical Center of Serbia, 11 000 Belgrade, Serbia; asretenovic77@gmail.com (A.S.); kecman.natalija@gmail.com (N.K.); majaperunicicjovanovic@yahoo.com (M.P.J.); biladr.jelena@gmail.com (J.B.); 2 Medical Faculty, University of Belgrade, 11 000 Belgrade, Serbiaukcs.hematologija.mijelom@gmail.com (R.M.); danijela.lekovic@yahoo.com (D.L.);; 3 Department for Hemato-Oncology, University Medical Center “Zvezdara”, 11 000 Belgrade, Serbia; nvukosavljevic93@gmail.com; 4 Center for Nuclear Medicine, University Clinical Center of Serbia, 11 000 Belgrade, Serbia; 5 Center for Magnetic Resonance Imaging, University Clinical Center of Serbia, 11 000 Belgrade, Serbia; 6 Institute of Statistics and Bioinformatics, Medical Faculty, University of Belgrade, 11 000 Belgrade, Serbia; zoran.bukumiric@med.bg.ac.rs; 7 Clinic of Hematology, Daily Hospital Department, University Clinical Center of Serbia, 11 000 Belgrade, Serbia; 8 Clinic of Cardiology, University Clinical Center of Serbia, 11 000 Belgrade, Serbia; 9 Clinic of Hematology, Department for Stem Cell Transplantation, University Clinical Center of Serbia, 11 000 Belgrade, Serbia

**Keywords:** AL amyloidosis, treatment, prognosis, real-world data

## Abstract

**Background:** Limited real-world data (RWD) may provide important information regarding diagnostic and treatment patterns in patients (pts) with AL Amyloidosis. The aim was to analyze the characteristics, treatment approach and clinical outcome of patients in the real-world settings. **Materials and Methods:** RWD of 60 pts diagnosed with AL amyloidosis were analyzed. Advanced cardiac involvement, Mayo Clinical Stage (CS) IIIa and IIIb, and Revised-Mayo CS III and IV, has been found in 26.7%, and 16.7%, or 33.3% and 16.7%, respectively. Bortezomib (Bz)-based regimens were applied in 36 pts (60%), and alkylating-based regimens in 24 pts (40%). In 8 pts (13.3%) treated initially with CyBorD induction, high-dose therapy with Melphalan and autologous stem cell transplantation (HDT + ASCT) was applied as the first line of treatment. **Results:** The overall response rate (ORR, ≥partial response) was achieved in 40 pts (70%). Patients treated with Bz-based induction followed by HDT + ASCT achieved significantly better hematologic (*p* = 0.001), cardiac (*p* = 0.004) and renal response rates (*p* = 0.002) in comparison to CyBorD or Alk-based regimens alone. There was no difference in PFS between those groups (*p* = 0.733), but patients treated with CyBorD + HDT + ASCT had significantly durable OS (*p* = 0.039). Univariate analysis pointed out the predictive influence of cardiac involvement (Mayo CS and Revised Mayo CS), ASCT eligibility, and hematologic, cardiac, renal and composite response rates. **Conclusions:** Advanced cardiac involvement and cardiac and hematologic response still retain adverse prognostic impact on the clinical outcome. Bz-based combinations significantly improved the survival of patients with AL amyloidosis, regardless of HDT + ASCT treatment.

## 1. Introduction

With an average worldwide incidence of 5.1–12.8 newly diagnosed patients/1.000.000 inhabitants annually, light chain amyloidosis (AL amyloidosis) is a rare plasma cell dyscrasia [1,2,3]. The present mainstream of AL amyloidosis treatment is focused on reducing the plasma cell secretion of free light chains and preventing further organ damage. The treatment choice is mainly based on the presence of cardiac involvement as a major limiting criterion for treatment with high-dose therapy and autologous stem cell transplantation [4,5]. Current recommendations indicate Bz-based regimens as the standard first-line treatment, preferably in combination with monoclonal anti-CD38 antibody, Daratumumab, followed by HDT + ASCT in accordance with transplant eligibility criteria, which are present in ≈20% newly diagnosed patients. The treatment approach of relapsed/refractory (R/R) AL amyloidosis, similarly to the treatment of R/R multiple myeloma, is based on the previously applied treatments and duration of response [6,7,8,9,10]. Considering that the antifibrillar drugs Birtamimab and Anselamimab failed to achieve the primary endpoint in clinical trials, clone-directed therapies remain the standard of care for AL amyloidosis patients [11,12]. Due to the rare characteristics of this disease, the RWD is accompanied by challenges in the diagnosis and treatment of AL amyloidosis [13,14].

The goal of this study was to analyze the prognostic significance of clinical characteristics and different staging indices in patients with AL amyloidosis treated outside of clinical trials, and their impact on treatment approach and follow-up in the RWD settings.

## 2. Materials and Methods

### 2.1. Patients

A total of 60 patients (pts) with AL amyloidosis, newly diagnosed during the period of time from January 2015 to July 2023, at the Ward for multiple myeloma and related plasma cell disorders, Clinic of Hematology, University Clinical Center of Serbia, were enrolled in this retrospective study. In the analyzed group, adult patients older than 18 years were included, with a minimum follow-up period and/or OS ≥ 6 months. The diagnosis and organ involvement criteria were based on the recommendations of the International Society of Amyloidosis (ISA) Working Group. The amyloid deposits were revealed by examination of tissue biopsies stained with Congo red and/or Thioflavin T, followed by immunohistochemical typing in abdominal fat tissue biopsy, bone marrow, gingiva, or affected organ biopsy, in the presence of monoclonal plasma cell proliferative disorder and amyloid-related systemic syndrome (affecting one or more organs) [15,16,17]. All pts with a localized form of AL amyloidosis were excluded from the study. Presence of specific chromosomal abnormalities [CAs: del (13q); IgH rearrangement; t (11;14); del (17p); amp (1q); del (1p)] were detected in 12 pts by applying interphase fluorescence in situ hybridization (iFISH) in 25 pts with ≥10% monoclonal plasma cells in the bone marrow, performed in accordance with the current International Myeloma Working Group recommendations and European Society for Medical Oncology Clinical Practice Guidelines. According to European Society of Cardiology Guidelines (2021); echocardiographic signs of cardiac amyloidosis included left ventricular wall thickness ≥ 12 mm in the presence of 2 following criteria: grade ≥ 2 of diastolic dysfunction, reduced tissue Doppler s’, e’ and a’ waves velocities (<5 cm/s), and decreased global longitudinal left ventricular strain (<−15%). Cardiac magnetic resonance criteria included 2 mandatory findings, such as diffuse subendocardial or transmural late gadolinium enhancement (LGE) and abnormal gadolinium kinetics, as well as increased extracellular volume (ECV ≥ 0.4%) if it was present [18]. The patients’ characteristics are summarized in Table 1.

Patients were staged in accordance with the cardiac risk models: Mayo 2004, Revised Mayo 2012 and European 2015 modification of Mayo 2004 [19,20,21]. Regarding renal impairment, patients were stratified by the Palladini model [15,22,23,24]. (Table 2).

### 2.2. Treatment

In accordance with the current presence of organ involvement and access to new treatment modalities at the time of diagnosis, patients (pts) were treated as follows: (1) Bz-based regimens (CyBorD) in 22 pts (36.7%); (2) CyBorD followed by HDT + ASCT in 8 pts (13.3%); (3) alkylating (Alk)-based chemotherapy in 22 pts (36.7%); and (4) Alk-based chemotherapy followed by HDT + ASCT in 2 pts (3.3%). In relapse, which was recorded in 19 pts (31.7%), patients were treated with alkylating agents (7/19 pts), Daratumumab-based immunochemotherapy (5/19 pts), Bortezomib (2/19 pts), and low-dose Lenalidomide (1/19 pts), while 4/19 pts died due to complications of heart failure before initiating the second-line treatment. Treatment response was evaluated according to hematological and organ response ISA-updated criteria and composite hematological and organ response (CHOR) criteria [25,26,27].

The study was performed according to the guidelines of the Declaration of Helsinki Principles and Good Clinical Practice.

### 2.3. Statistical Analysis

The clinical characteristics of patients were analyzed using descriptive statistics. Statistical analysis included the following testing methods: *t*-test, Mann–Whitney test, chi-square test, and Fisher’s exact probability test. OS was the main endpoint of the present study, and PFS was included as an additional endpoint. OS was measured from the diagnosis until the last follow-up or death, while PFS was measured from the diagnosis until the last follow-up or the occurrence of an event (progression, relapse, or death). Time to event curves were plotted with the Kaplan and Meier method, and comparisons among groups were made using the Log Rank and Kruskal–Wallis test. A cross-tab test was performed to identify the adverse group of patients. Statistical significance was set at *p* ≤ 0.05. All statistical analyses were conducted using IBM SPSS Statistics 22.0 (R software environment v4.3.0; R Core Team 2023).

## 3. Results

### 3.1. Treatment Response

Overall hematologic response rate (ORR, ≥partial response, PR) was achieved in 40 pts (70%): complete response (CR) was recorded in 8 pts (13.3%), very good partial response (VGPR) in 11 pts (18.3%), and PR in 21 pts (35%). Considering organ response, 33 pts (62.3%) had cardiac response, 38 pts (63.3%) achieved renal response, and liver response was recorded in 6 pts (66.7%). Complete cardiac response was observed in 8 pts (15.7%), very good partial response in 6 pts (11.8%), and partial response in 19 pts (37.2%). Complete renal response was present in 11 pts (20.7%), very good partial response in 3 pts (5.6%), and partial response in 24 pts (45.2%). Composite response (CHOR 1) was achieved in 41 pts (68.3%) (Table 3). Patients in the CHOR 2 group were characterized with significantly higher cardiac involvement according to Mayo CS (68.4% of the patients were in IIIa and IIIb CS; *p* = 0.002) and Revised-Mayo CS (73.7% in Revised-Mayo CS III and IV; *p* = 0.002).

Patients treated with Bz-based induction followed by HDT + ASCT had a significantly better response rate (≥PR) in comparison to CyBorD alone (8 pts, 100% vs. 21 pts, 75%; *p* = 0.001). Additionally, patients treated with CyBorD alone had a significantly better response rate in comparison to Alk-based regimens alone (21 pts, 75% vs. 9 pts, 40.9%; *p* = 0.001). Patients treated with CyBorD + HDT + ASCT achieved the highest rate of CR and VGPR in comparison to CyBorD alone and Alk-based regimens alone (75% vs. 28.6% vs. 13.6%, respectively). The highest level of overall cardiac response was observed in the CyBorD + HDT + ASCT group (7 pts, 100%), while in the CyBorD group overall cardiac response was confirmed in 17 pts (70.8%), and in the Alk-based group in 8 pts (38.1%), with statistical significance between groups (*p* = 0.004). Overall renal response followed a similar trend to a cardiac response, with 8 pts (100%) in the CyBorD + HDT + ASCT group vs. 21 pts (80.8%) in the CyBorD group vs. 8 pts (42.1%) in the Alk-based group (*p* = 0.002). The examined groups did not differ significantly in terms of age (*p* = 0.318), sex distribution (*p* = 0.874), Mayo clinical stage (*p* = 0.147), Revised-Mayo clinical stage (*p* = 0.117), or renal stage (*p* = 0.899). Both of the two patients treated with Alk-based regimens followed by ASCT achieved complete hematologic response accompanied by complete organ response.

### 3.2. Survival Analysis

Two patients treated with Alk-based regimens followed by ASCT were excluded from the survival analysis. The PFS for these two patients was 37 months and 58 months, respectively, while OS was 48 months and 63 months, respectively.

**Figure 1:**
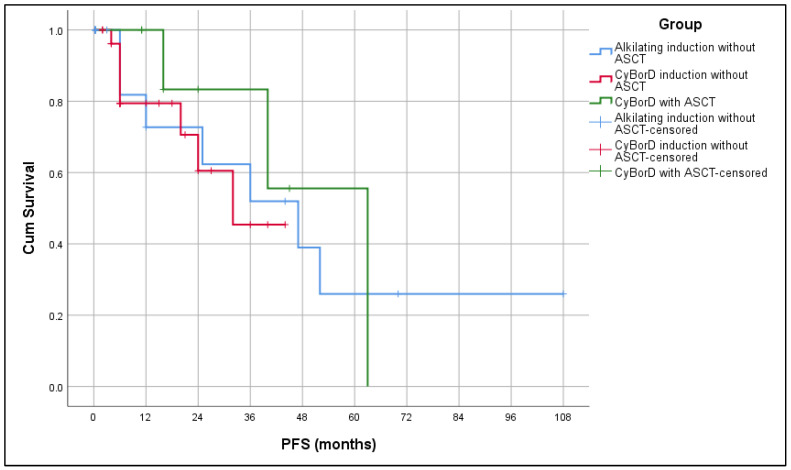
Kaplan–Meier analysis of progression-free survival (PFS) in accordance with applied treatment.

**Figure 2:**
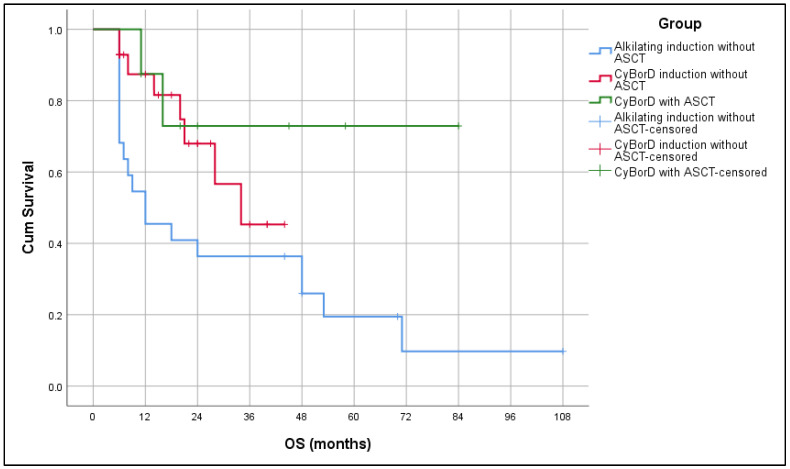
Kaplan–Meier analysis of overall survival (OS) in accordance with applied treatment.

The median PFS by Kaplan–Meier analysis for the whole group was 34 months (range: range: 13–54 months), while the median OS for the whole group was 48 months (range: 21.6–74.3 months). Median OS in the Alk-based group was 12 months, and in the CyBorD group 34 months, while in CyBorD + ASCT median OS was not reached. While a difference in PFS was not observed between groups (*p* = 0.733) (Figure 1), there was a statistically significant difference in OS (*p* = 0.039). The CyBorD + ASCT group had the longest survival (Figure 2).

Univariate analysis pointed out the impact of the following patients’ characteristics on the OS: clinical stage according to Mayo CS and Revised Mayo stratification, hematologic, cardiac, renal and composite response, while the influence of ASCT eligibility had borderline statistical significance (Table 4). OS was not influenced by the age (*p* = 0.126; HR 1.03 (0.99–1.07)), gender (*p* = 0.584; HR 0.81 (0.39–1.71)), number of affected organs (*p* = 0.564; HR 1.14 (0.73–1.78)), elevated LDH (*p* = 0.317; HR 1.45 (0.68–3.05)), renal CS (*p* = 0.262; HR 0.73 (0.42–1.26), or infiltration of the bone marrow with ≥10% plasma cells (*p* = 0.368; HR 1.41 (0.66–2.98)).

## 4. Discussion

Early recognition and diagnostics of patients with AL amyloidosis are still challenging [28,29]. Due to the rarity of the disease, merging RWD from different countries and centers provides information of essential importance regarding characteristics of the disease, treatment approaches, patient follow-up, and outcomes outside of clinical trials [13,30]. In comparison to the patients’ data from the EMN23 observational RWD study, in our cohort, the majority of the patients had a higher frequency of cardiac (85%) and renal (88.3%) involvement, implying the necessity of earlier recognition and diagnostics. Regarding the diagnostics, in the majority of our patients, amyloid deposition was present in fat tissue biopsies (70%) and/or bone marrow biopsies (35%) as the two most common sites, in accordance with the published data, accompanied by findings of verified clonal immunoglobulin lambda light chain disease in 42 pts (70%) [31]. Echocardiography was performed in all patients, with signs of cardiac amyloidosis found in 66.1% of patients. However, with magnetic resonance imaging, a significantly more sensitive tool for cardiac amyloidosis, amyloid depositions were found in 86.7% patients in the analyzed group. Conducting the differential diagnostics of transthyretin (ATTR) cardiac amyloidosis, technetium pyrophosphate scintigraphy was performed in 12 patients, resulting in negative uptake of tracer (Perugini score 0) in all 12 patients, which is in concordance with scintigraphy findings in patients with AL amyloidosis [32,33]. A high proportion (43.4%) of our patients had advanced cardiac involvement (Mayo CS IIIa and IIIb), probably as a result of late diagnosis, which correlates with the results of the larger study groups (44.2%) [30].

Considering Daratumumab’s limited availability as the first-line treatment at the time of conducting this analysis, a Bortezomib-based induction regimen such as CyBorD was the most commonly used in the group of transplant-eligible patients and patients with potentially reversible contraindications for treatment with HDT + ASCT. Historically, alkylating-based regimens were the standard of care for a long period of time for transplant-ineligible patients with cardiac involvement, before the introduction of new treatment modalities as Bortezomib and especially anti-CD38 monoclonal antibody Daratumumab [34]. In accordance with the ISA recommendation, two-thirds of our patients were treated with Bortezomib-based combinations in the first line of treatment [9,10]. The selected treatment approach was chosen based on the standard of care at the time of diagnosis and accessibility of new treatment modalities, as well as on the extent of the involvement of affected organs, age, and comorbidities.

Regarding the treatment response, a higher frequency of ORR (≥PR) was achieved in patients treated with Bortezomib-based combos, with or without HD Melphalan and ASCT, in comparison to the low response rate in the group treated without Bortezomib (CyBorD + HDT + ASCT: ORR 100%, CyBorD: ORR 75% vs. Alk-based group without HDT + ASCT: ORR 40.9%), which correlates to the EMN23 treatment results [30]. Similar treatment results were observed in the European collaborative study previously published in 2015 by Palladini et al. [21]. Regarding OS, our study demonstrated the superiority of Bortezomib regimens (with or without HDT + ASCT consolidation) over standard, conventional chemotherapy (*p* = 0.039), especially in the group with HDT + ASCT consolidation after Bortezomib-based induction with the longest OS. There was no difference in PFS between groups in our study (*p* = 0.733), probably due to the shorter follow-up period of the patients treated with Bortezomib-based regimens in circumstances of limited accessibility to such treatment.

Currently, immunochemotherapy with anti-CD38 antibody Daratumumab represents the optimal first-line treatment approach based on the highest rate of hematologic CR and VGPR, as well as deep organ responses, based on the results of the ANDROMEDA trial [34]. If immunochemotherapy is available as the first-line treatment, in the case of CR achievement, HDT followed by ASCT could be delayed to the first suggestion of hematologic relapse [10]. High-dose Melphalan and ASCT, with or without induction treatment, remains the standard first-line treatment in a modest number of patients without advanced cardiac involvement if immunochemotherapy is unavailable [35]. Pre-transplant induction therapy can decrease the risk of relapse, and in combination with ASCT it can greatly increase the rate of CR up to 60%, with more than 50% patients with an OS longer than 15 years [36]. The data published by Sidana et al. additionally pointed out the importance of the Melphalan full dose (200 mg/m^2^) for longer OS [37]. In the analyzed group of patients, patients treated with CyBorD + HDT + ASCT had statistically longer OS in comparison to both non-transplant groups (*p* = 0.039). However, transplant eligibility is strongly influenced by the cardiac stage of disease as the main limiting factor for intense treatment, consequently leading to longer OS for patients with an early cardiac stage despite the treatment modality. Furthermore, the achievement of early hematological response accompanied by a decrease in amyloidogenic free light chains is mandatory for preventing further cardiac damage [10,31].

Due to multi-organ involvement, the necessity of including organ response criteria has been raised. The first composite model that incorporates both hematologic and organ response was introduced by Sidana et al. [25]. The score pointed out that patients in the favorable CHOR 1 group had significantly better outcomes compared to the CHOR 2 group, as was confirmed in our patients as well (HR 3.92, 95% CI 3.86–20.61, *p* < 0.01). Clinical stratification in accordance with the Mayo and Revised-Mayo staging system, followed by cardiac response at the end of induction, strongly influenced the OS in our study group, as well as the poor treatment outcome. The majority of patients in the CHOR 2 group had advanced cardiac involvement (68.4% in Mayo CS IIIa and IIIb CS; 73.7% in R-Mayo CS III and IV), indicating that cardiac involvement remains the leading prognostic factor [3,14,38].

## 5. Conclusions

In summary, AL amyloidosis presents a unique diagnostic and treatment challenge due to its diverse clinical manifestations and organ involvement, particularly in the context of cardiac disease. Bortezomib-based treatment, followed by HDT and ASCT and achievement of hematologic and organ response, retains its prognostic impact on the overall survival if treatment with anti-CD38 immunochemotherapy is unavailable.

## Figures and Tables

**Table 1:** Distribution of patients in accordance with demographic, laboratory and imaging characteristics.

Characteristics	
Gender—no (%)	
Male	30 (50%)
Female	30 (50%)
Age	
Median (range)—yrs	59 (29–77)
AL isotype—no (%)	
Lambda (λ) light chain M protein	42 (70%)
Organ involvement—no (%)	
Kidney	53 (88.3%)
Heart	51 (85%)
Liver	12 (20%)
Alternate sites	15 (25%)
Proven amyloid deposits—no (%)	
Affected organ	11 (18.3%)
Abdominal fat tissue	42 (70%)
Bone marrow	21 (35%)
Gingiva	2 (3.3%)
Laboratory characteristics—no (%)	
LDH elevation (>460 U/L)	27 (45%)
Anemia (hemoglobin < 100 g/L)	10 (16.7%)
Biomarkers of cardiac involvement—no (%)	
hsTroponin T (>14 ng/L)	46 (77%)
BNP (>100 ng/L)	31 (67%)
NT-proBNP (>125 ng/L)	55 (92%)
NYHA class	
NYHA I-II	20 (33.3%)
NYHA III	25 (41.6%)
NYHA IV	15 (25%)
Renal impairment—no (%)	
Nephrotic proteinuria (≥3.5 gr/24 h)	33 (60%)
eGFR < 60 mL/min/1.73 m^2^	25 (41.7%)
Hemodialysis support	5 (8.3%)
Imaging of cardiac amyloidosis—no (%)	
Echocardiography	39 (66.1%)
Cardiac magnetic resonance	13 (86.7%)
iFISH analysis—no (%)	
CAs	12 (48%)

**Table 2:** Distribution of patients in accordance with staging systems.

Mayo 2004 Model—No (%)	
1	13 (27.1%)
2	21 (35%)
3	26 (43.4%)
Revised Mayo 2012 model—no (%)	
1	19 (31.7%)
2	11 (18.3%)
3	20 (33.3%)
4	10 (16.7%)
European 2015 modification of Mayo 2004 model—no (%)	
1	13 (27.1%)
2	21 (35%)
3a	16 (26.7%)
3b	10 (16.7%)
Palladini renal clinical stage—no (%)	
1	23 (37.1%)
2	25 (41.7%)
3	12 (20%)

**Table 3:** Treatment response to induction therapy.

Hematologic Response—No (%)	
Complete response (CR)	8 (13.3%)
Very good partial response (VGPR)	11 (18.3%)
Partial response (PR)	21 (35%)
Organ response—no (%)	
Overall cardiac response	33 (62.3%)
Complete cardiac response	8 (15.7%)
Very good partial cardiac response	6 (11.8%)
Partial cardiac response	19 (37.2%)
Renal response	38 (63.3%)
Complete renal response	11(20.7%)
Very good partial renal response	3 (5.6%)
Partial renal response	24 (45.2%)
Liver response	6 (66.7%)
Composite response (CHOR)—no (%)	
CHOR 1	41 (68.3%)
CHOR 2	19 (31.7%)

**Table 4:** Prognostic factors of impact on the OS by the univariate model.

Covariates	Hazard Ratio, 95% CI	*p* Value
Mayo CS	1.79 (1.17–2.73)	*p* < 0.01
Revised-Mayo CS	1.80 (1.23–2.64)	*p* < 0.01
HDT + ASCT treatment	0.29 (0.08–1.00)	*p* = 0.05
Hematological response	0.15 (0.07–0.35)	*p* < 0.01
Cardiac response	0.13 (0.05–0.31)	*p* < 0.01
Renal response	0.17 (0.07–0.41)	*p* < 0.01
CHOR	3.92 (3.86–20.61)	*p* < 0.01

## Data Availability

The data that support the findings of this study are not openly available due to reasons of sensitivity and are available from the corresponding author upon reasonable request. Data are located in controlled access data storage at the Clinic of Hematology, University Clinical Centre of Serbia.
